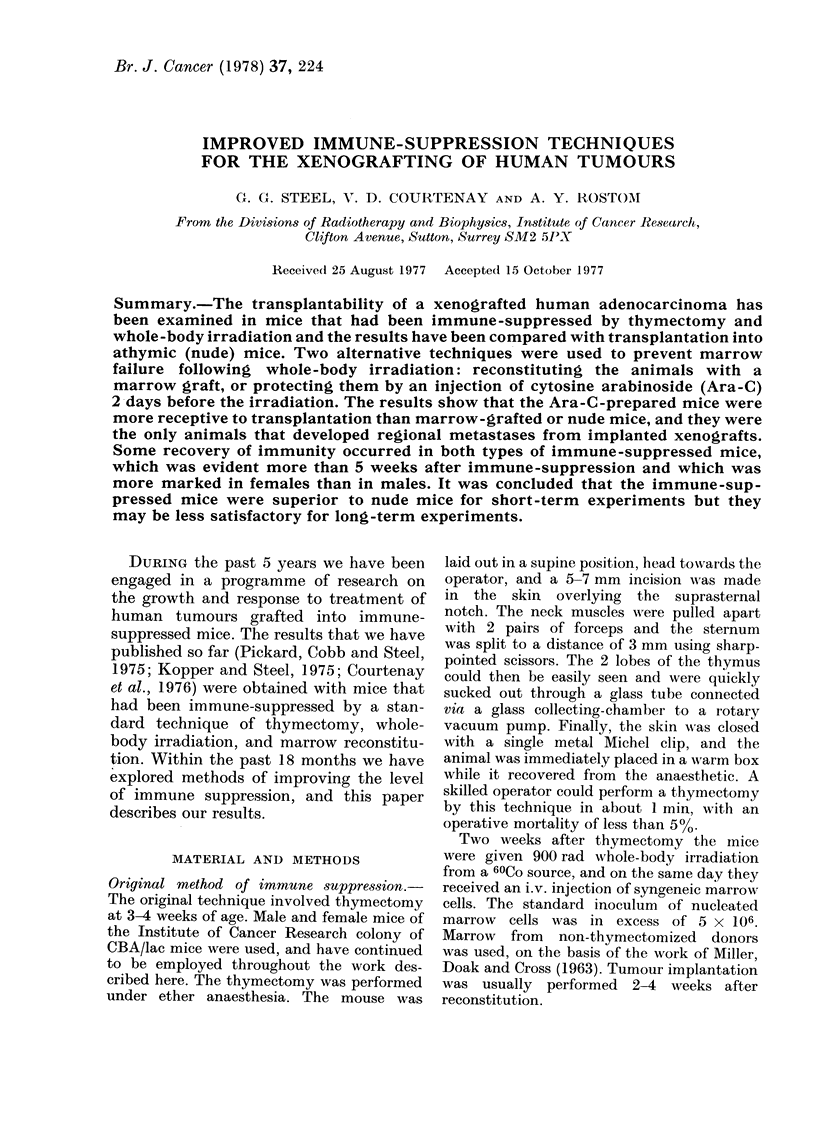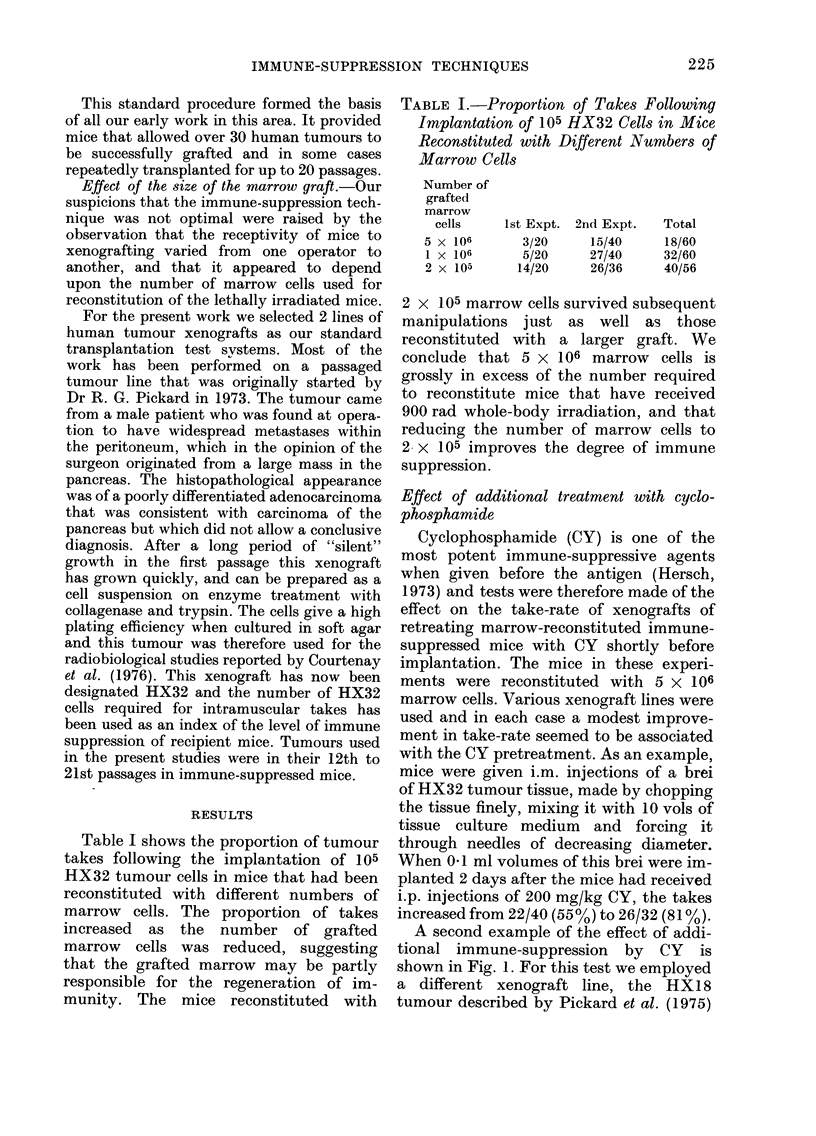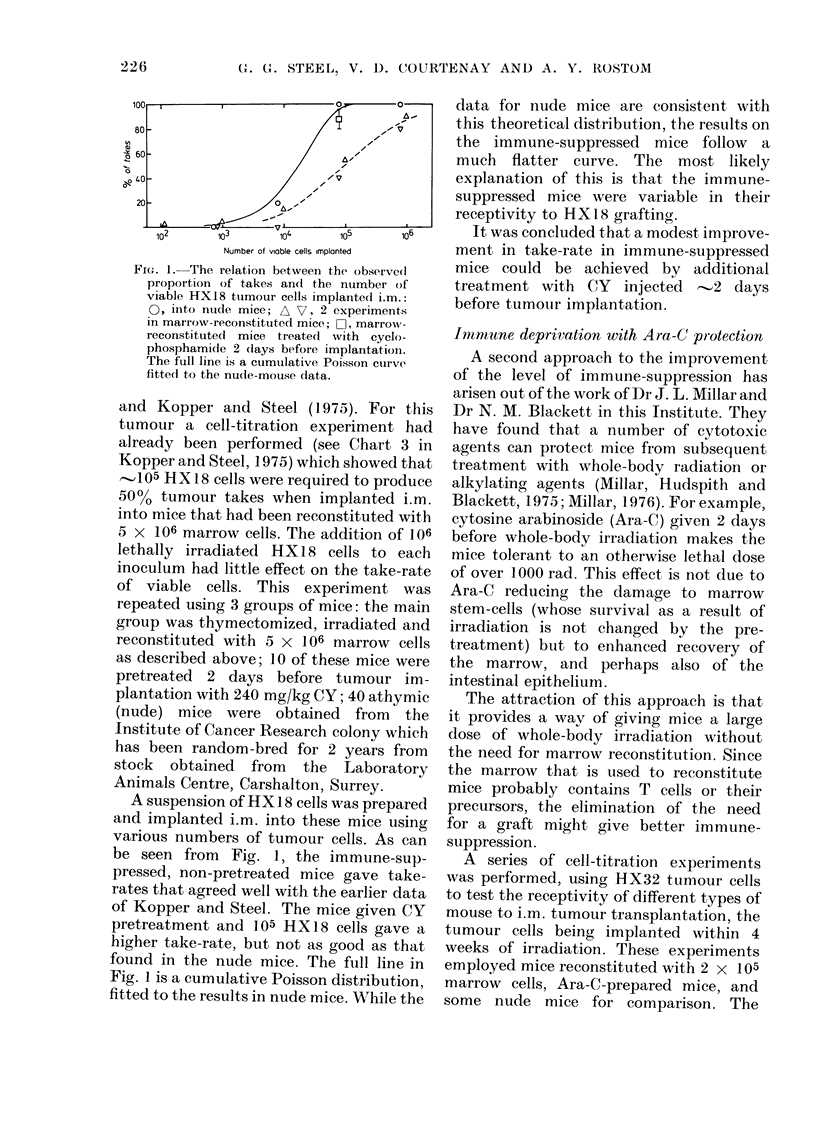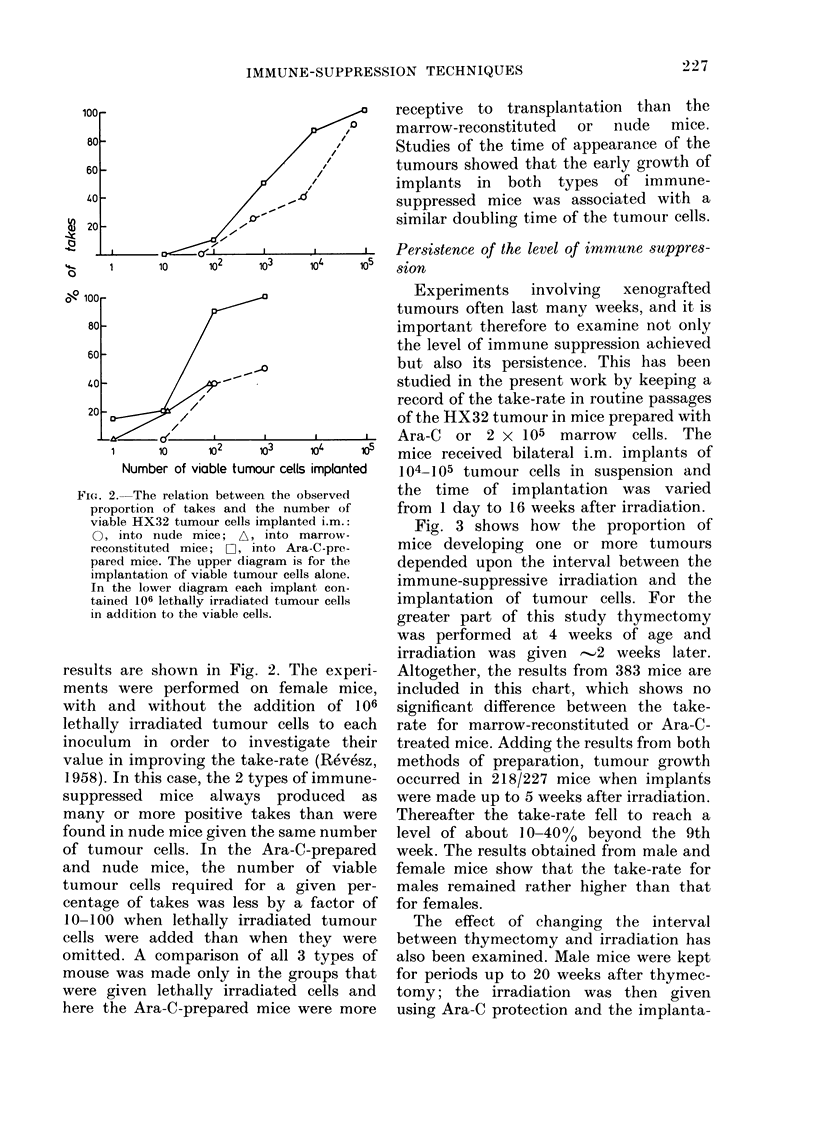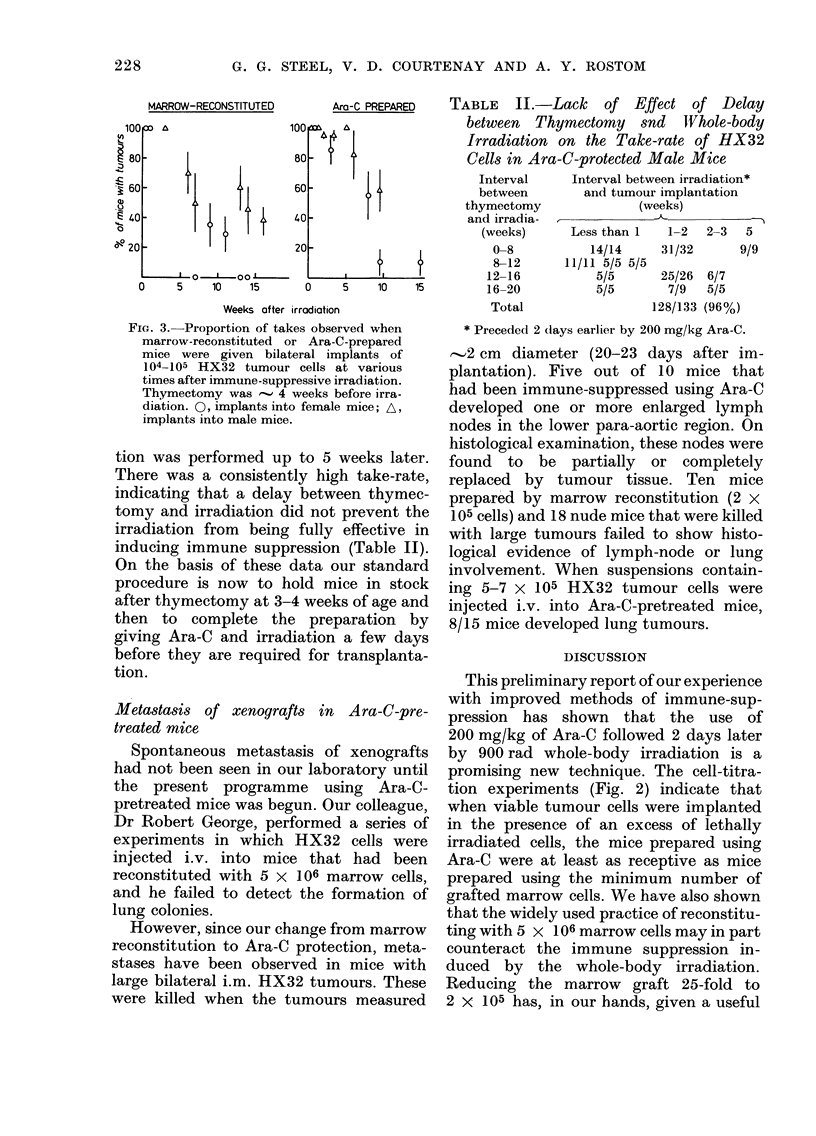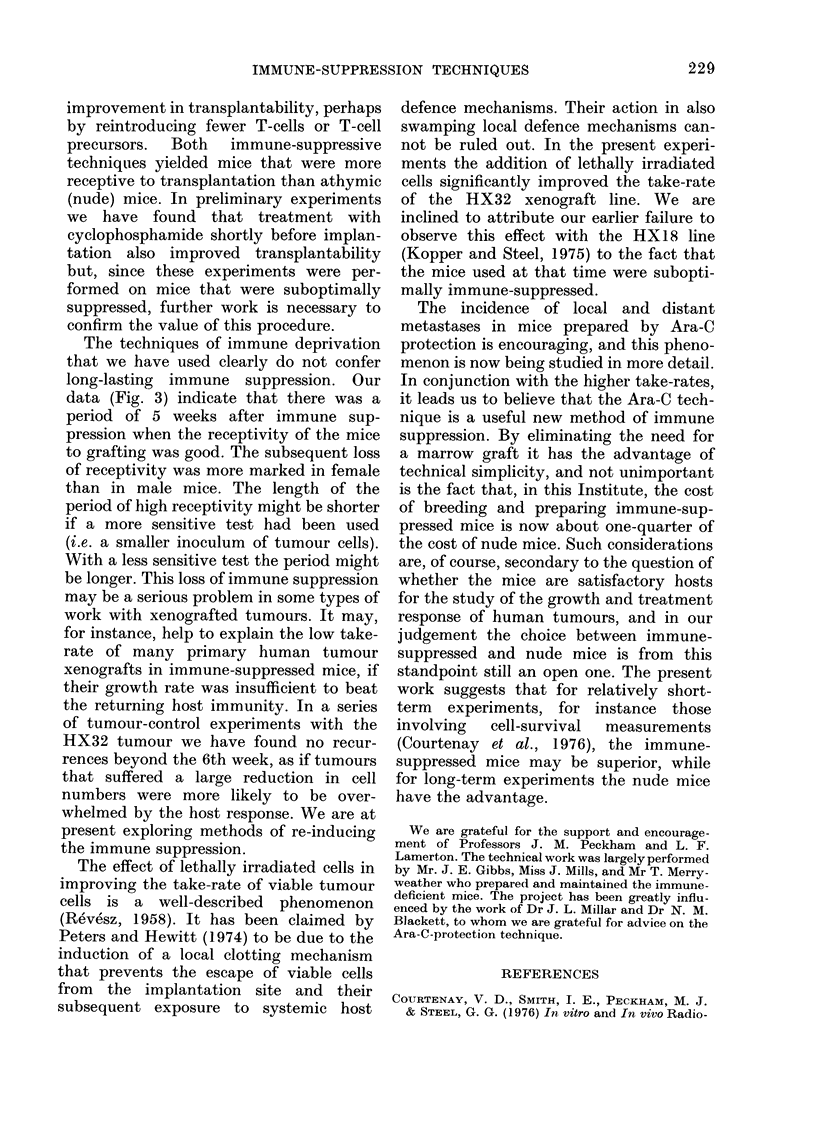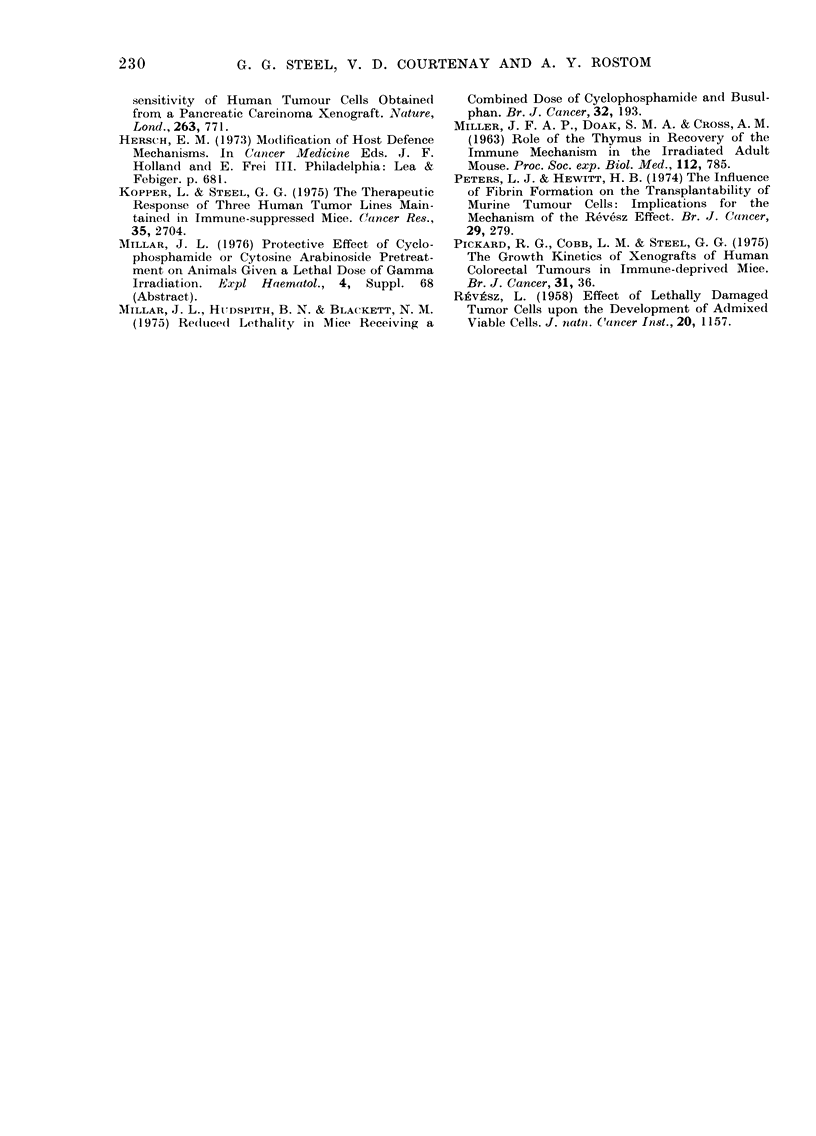# Improved immune-suppression techniques for the exongrafting of human tumours.

**DOI:** 10.1038/bjc.1978.30

**Published:** 1978-02

**Authors:** G. G. Steel, V. D. Courtenay, A. Y. Rostom

## Abstract

The transplantability of a xenografted human adenocarcinoma has been examined in mice that had been immune-suppressed by thymectomy and whole-body irradiation and the results have been compared with transplantation into athymic (nude) mice. Two alternative techniques were used to prevent marrow failure following whole-body irradiation: reconstituting the animals with a marrow graft, or protecting them by an injection of cytosine arabinoside (Ara-C) 2 days before the irradiation. The results show that the Ara-C-prepared mice were more receptive to transplantation than marrow-grafted or nude mice, and they were the only animals that developed regional metastases from implanted xenografts. Some recovery of immunity occurred in both types of immune-suppressed mice, which was evident more than 5 weeks after immune-suppression and which was more marked in females than in males. It was concluded that the immune-suppressed mice were superior to nude mice for short-term experiments but they may be less satisfactory for long-term experiments.


*Br. J. Cancer* (1978) **37**, 224

### IMPROVED IMMUNE-SUPPRESSION TECHNIQUES FOR THE XENOGRAFTING OF HUMAN TUMOURS

G. G. STEEL, V. D. COURTENAY AND A. Y. ROSTOM

*From the Divisions of Radiotherapy and Biophysics, Institute of Cancer Research, Clifton Avenue, Sutton, Surrey SM2 5PX*

Received 25 August 1977 Accepted 15 October 1977

**Summary.—The transplantability of a xenografted human adenocarcinoma has been examined in mice that had been immune-suppressed by thymectomy and whole-body irradiation and the results have been compared with transplantation into athymic (nude) mice. Two alternative techniques were used to prevent marrow failure following whole-body irradiation: reconstituting the animals with a marrow graft, or protecting them by an injection of cytosine arabinoside (Ara-C) 2 days before the irradiation. The results show that the Ara-C-prepared mice were more receptive to transplantation than marrow-grafted or nude mice, and they were the only animals that developed regional metastases from implanted xenografts. Some recovery of immunity occurred in both types of immune-suppressed mice, which was evident more than 5 weeks after immune-suppression and which was more marked in females than in males. It was concluded that the immune-suppressed mice were superior to nude mice for short-term experiments but they may be less satisfactory for long-term experiments.**

DURING the past 5 years we have been engaged in a programme of research on the growth and response to treatment of human tumours grafted into immune-suppressed mice. The results that we have published so far (Pickard, Cobb and Steel, 1975; Kopper and Steel, 1975; Courtenay *et al.*, 1976) were obtained with mice that had been immune-suppressed by a standard technique of thymectomy, whole-body irradiation, and marrow reconstitution. Within the past 18 months we have explored methods of improving the level of immune suppression, and this paper describes our results.

### MATERIAL AND METHODS

*Original method of immune suppression.*—The original technique involved thymectomy at 3–4 weeks of age. Male and female mice of the Institute of Cancer Research colony of CBA/lac mice were used, and have continued to be employed throughout the work described here. The thymectomy was performed under ether anaesthesia. The mouse was laid out in a supine position, head towards the operator, and a 5–7 mm incision was made in the skin overlying the suprasternal notch. The neck muscles were pulled apart with 2 pairs of forceps and the sternum was split to a distance of 3 mm using sharp-pointed scissors. The 2 lobes of the thymus could then be easily seen and were quickly sucked out through a glass tube connected *via* a glass collecting-chamber to a rotary vacuum pump. Finally, the skin was closed with a single metal Michel clip, and the animal was immediately placed in a warm box while it recovered from the anaesthetic. A skilled operator could perform a thymectomy by this technique in about 1 min, with an operative mortality of less than 5%.

Two weeks after thymectomy the mice were given 900 rad whole-body irradiation from a $^{60}$Co source, and on the same day they received an i.v. injection of syngeneic marrow cells. The standard inoculum of nucleated marrow cells was in excess of $5 \times 10^6$. Marrow from non-thymectomized donors was used, on the basis of the work of Miller, Doak and Cross (1963). Tumour implantation was usually performed 2–4 weeks after reconstitution.

This standard procedure formed the basis of all our early work in this area. It provided mice that allowed over 30 human tumours to be successfully grafted and in some cases repeatedly transplanted for up to 20 passages.

*Effect of the size of the marrow graft.*—Our suspicions that the immune-suppression technique was not optimal were raised by the observation that the receptivity of mice to xenografting varied from one operator to another, and that it appeared to depend upon the number of marrow cells used for reconstitution of the lethally irradiated mice.

For the present work we selected 2 lines of human tumour xenografts as our standard transplantation test systems. Most of the work has been performed on a passaged tumour line that was originally started by Dr R. G. Pickard in 1973. The tumour came from a male patient who was found at operation to have widespread metastases within the peritoneum, which in the opinion of the surgeon originated from a large mass in the pancreas. The histopathological appearance was of a poorly differentiated adenocarcinoma that was consistent with carcinoma of the pancreas but which did not allow a conclusive diagnosis. After a long period of "silent" growth in the first passage this xenograft has grown quickly, and can be prepared as a cell suspension on enzyme treatment with collagenase and trypsin. The cells give a high plating efficiency when cultured in soft agar and this tumour was therefore used for the radiobiological studies reported by Courtenay *et al.* (1976). This xenograft has now been designated HX32 and the number of HX32 cells required for intramuscular takes has been used as an index of the level of immune suppression of recipient mice. Tumours used in the present studies were in their 12th to 21st passages in immune-suppressed mice.

### RESULTS

Table I shows the proportion of tumour takes following the implantation of $10^5$ HX32 tumour cells in mice that had been reconstituted with different numbers of marrow cells. The proportion of takes increased as the number of grafted marrow cells was reduced, suggesting that the grafted marrow may be partly responsible for the regeneration of immunity. The mice reconstituted with

TABLE I.—*Proportion of Takes Following Implantation of* $10^5$ *HX32 Cells in Mice Reconstituted with Different Numbers of Marrow Cells*

| Number of grafted marrow cells | 1st Expt. | 2nd Expt. | Total |
|---|---|---|---|
| $5 \times 10^6$ | 3/20 | 15/40 | 18/60 |
| $1 \times 10^6$ | 5/20 | 27/40 | 32/60 |
| $2 \times 10^5$ | 14/20 | 26/36 | 40/56 |

$2 \times 10^5$ marrow cells survived subsequent manipulations just as well as those reconstituted with a larger graft. We conclude that $5 \times 10^6$ marrow cells is grossly in excess of the number required to reconstitute mice that have received 900 rad whole-body irradiation, and that reducing the number of marrow cells to $2 \times 10^5$ improves the degree of immune suppression.

### *Effect of additional treatment with cyclophosphamide*

Cyclophosphamide (CY) is one of the most potent immune-suppressive agents when given before the antigen (Hersch, 1973) and tests were therefore made of the effect on the take-rate of xenografts of retreating marrow-reconstituted immune-suppressed mice with CY shortly before implantation. The mice in these experiments were reconstituted with $5 \times 10^6$ marrow cells. Various xenograft lines were used and in each case a modest improvement in take-rate seemed to be associated with the CY pretreatment. As an example, mice were given i.m. injections of a brei of HX32 tumour tissue, made by chopping the tissue finely, mixing it with 10 vols of tissue culture medium and forcing it through needles of decreasing diameter. When 0·1 ml volumes of this brei were implanted 2 days after the mice had received i.p. injections of 200 mg/kg CY, the takes increased from 22/40 (55%) to 26/32 (81%).

A second example of the effect of additional immune-suppression by CY is shown in Fig. 1. For this test we employed a different xenograft line, the HX18 tumour described by Pickard *et al.* (1975)

FIG. 1.—The relation between the observed proportion of takes and the number of viable HX18 tumour cells implanted i.m.: ○, into nude mice; △ ▽, 2 experiments in marrow-reconstituted mice; □, marrow-reconstituted mice treated with cyclophosphamide 2 days before implantation. The full line is a cumulative Poisson curve fitted to the nude-mouse data.

and Kopper and Steel (1975). For this tumour a cell-titration experiment had already been performed (see Chart 3 in Kopper and Steel, 1975) which showed that $\sim 10^5$ HX18 cells were required to produce 50% tumour takes when implanted i.m. into mice that had been reconstituted with $5 \times 10^6$ marrow cells. The addition of $10^6$ lethally irradiated HX18 cells to each inoculum had little effect on the take-rate of viable cells. This experiment was repeated using 3 groups of mice: the main group was thymectomized, irradiated and reconstituted with $5 \times 10^6$ marrow cells as described above; 10 of these mice were pretreated 2 days before tumour implantation with 240 mg/kg CY; 40 athymic (nude) mice were obtained from the Institute of Cancer Research colony which has been random-bred for 2 years from stock obtained from the Laboratory Animals Centre, Carshalton, Surrey.

A suspension of HX18 cells was prepared and implanted i.m. into these mice using various numbers of tumour cells. As can be seen from Fig. 1, the immune-suppressed, non-pretreated mice gave take-rates that agreed well with the earlier data of Kopper and Steel. The mice given CY pretreatment and $10^5$ HX18 cells gave a higher take-rate, but not as good as that found in the nude mice. The full line in Fig. 1 is a cumulative Poisson distribution, fitted to the results in nude mice. While the data for nude mice are consistent with this theoretical distribution, the results on the immune-suppressed mice follow a much flatter curve. The most likely explanation of this is that the immune-suppressed mice were variable in their receptivity to HX18 grafting.

It was concluded that a modest improvement in take-rate in immune-suppressed mice could be achieved by additional treatment with CY injected $\sim 2$ days before tumour implantation.

*Immune deprivation with Ara-C protection*

A second approach to the improvement of the level of immune-suppression has arisen out of the work of Dr J. L. Millar and Dr N. M. Blackett in this Institute. They have found that a number of cytotoxic agents can protect mice from subsequent treatment with whole-body radiation or alkylating agents (Millar, Hudspith and Blackett, 1975; Millar, 1976). For example, cytosine arabinoside (Ara-C) given 2 days before whole-body irradiation makes the mice tolerant to an otherwise lethal dose of over 1000 rad. This effect is not due to Ara-C reducing the damage to marrow stem-cells (whose survival as a result of irradiation is not changed by the pretreatment) but to enhanced recovery of the marrow, and perhaps also of the intestinal epithelium.

The attraction of this approach is that it provides a way of giving mice a large dose of whole-body irradiation without the need for marrow reconstitution. Since the marrow that is used to reconstitute mice probably contains T cells or their precursors, the elimination of the need for a graft might give better immune-suppression.

A series of cell-titration experiments was performed, using HX32 tumour cells to test the receptivity of different types of mouse to i.m. tumour transplantation, the tumour cells being implanted within 4 weeks of irradiation. These experiments employed mice reconstituted with $2 \times 10^5$ marrow cells, Ara-C-prepared mice, and some nude mice for comparison. The

FIG. 2.—The relation between the observed proportion of takes and the number of viable HX32 tumour cells implanted i.m.: ○, into nude mice; △, into marrow-reconstituted mice; □, into Ara-C-prepared mice. The upper diagram is for the implantation of viable tumour cells alone. In the lower diagram each implant contained $10^6$ lethally irradiated tumour cells in addition to the viable cells.

results are shown in Fig. 2. The experiments were performed on female mice, with and without the addition of $10^6$ lethally irradiated tumour cells to each inoculum in order to investigate their value in improving the take-rate (Révész, 1958). In this case, the 2 types of immune-suppressed mice always produced as many or more positive takes than were found in nude mice given the same number of tumour cells. In the Ara-C-prepared and nude mice, the number of viable tumour cells required for a given percentage of takes was less by a factor of 10–100 when lethally irradiated tumour cells were added than when they were omitted. A comparison of all 3 types of mouse was made only in the groups that were given lethally irradiated cells and here the Ara-C-prepared mice were more receptive to transplantation than the marrow-reconstituted or nude mice. Studies of the time of appearance of the tumours showed that the early growth of implants in both types of immune-suppressed mice was associated with a similar doubling time of the tumour cells.

*Persistence of the level of immune suppression*

Experiments involving xenografted tumours often last many weeks, and it is important therefore to examine not only the level of immune suppression achieved but also its persistence. This has been studied in the present work by keeping a record of the take-rate in routine passages of the HX32 tumour in mice prepared with Ara-C or $2 \times 10^5$ marrow cells. The mice received bilateral i.m. implants of $10^4$–$10^5$ tumour cells in suspension and the time of implantation was varied from 1 day to 16 weeks after irradiation.

Fig. 3 shows how the proportion of mice developing one or more tumours depended upon the interval between the immune-suppressive irradiation and the implantation of tumour cells. For the greater part of this study thymectomy was performed at 4 weeks of age and irradiation was given $\sim$2 weeks later. Altogether, the results from 383 mice are included in this chart, which shows no significant difference between the take-rate for marrow-reconstituted or Ara-C-treated mice. Adding the results from both methods of preparation, tumour growth occurred in 218/227 mice when implants were made up to 5 weeks after irradiation. Thereafter the take-rate fell to reach a level of about 10–40% beyond the 9th week. The results obtained from male and female mice show that the take-rate for males remained rather higher than that for females.

The effect of changing the interval between thymectomy and irradiation has also been examined. Male mice were kept for periods up to 20 weeks after thymectomy; the irradiation was then given using Ara-C protection and the implanta-

FIG. 3.—Proportion of takes observed when marrow-reconstituted or Ara-C-prepared mice were given bilateral implants of $10^4$–$10^5$ HX32 tumour cells at various times after immune-suppressive irradiation. Thymectomy was ~ 4 weeks before irradiation. ○, implants into female mice; △, implants into male mice.

tion was performed up to 5 weeks later. There was a consistently high take-rate, indicating that a delay between thymectomy and irradiation did not prevent the irradiation from being fully effective in inducing immune suppression (Table II). On the basis of these data our standard procedure is now to hold mice in stock after thymectomy at 3–4 weeks of age and then to complete the preparation by giving Ara-C and irradiation a few days before they are required for transplantation.

### *Metastasis of xenografts in Ara-C-pretreated mice*

Spontaneous metastasis of xenografts had not been seen in our laboratory until the present programme using Ara-C-pretreated mice was begun. Our colleague, Dr Robert George, performed a series of experiments in which HX32 cells were injected i.v. into mice that had been reconstituted with 5 × $10^6$ marrow cells, and he failed to detect the formation of lung colonies.

However, since our change from marrow reconstitution to Ara-C protection, metastases have been observed in mice with large bilateral i.m. HX32 tumours. These were killed when the tumours measured

TABLE II.—*Lack of Effect of Delay between Thymectomy snd Whole-body Irradiation on the Take-rate of HX32 Cells in Ara-C-protected Male Mice*

| Interval between thymectomy and irradia- (weeks) | Interval between irradiation* and tumour implantation (weeks) | | | |
|---|---|---|---|---|
| | Less than 1 | 1–2 | 2–3 | 5 |
| 0–8 | 14/14 | 31/32 | | 9/9 |
| 8–12 | 11/11 5/5 5/5 | | | |
| 12–16 | 5/5 | 25/26 | 6/7 | |
| 16–20 | 5/5 | 7/9 | 5/5 | |
| Total | | 128/133 (96%) | | |

* Preceded 2 days earlier by 200 mg/kg Ara-C.

~2 cm diameter (20–23 days after implantation). Five out of 10 mice that had been immune-suppressed using Ara-C developed one or more enlarged lymph nodes in the lower para-aortic region. On histological examination, these nodes were found to be partially or completely replaced by tumour tissue. Ten mice prepared by marrow reconstitution (2 × $10^5$ cells) and 18 nude mice that were killed with large tumours failed to show histological evidence of lymph-node or lung involvement. When suspensions containing 5–7 × $10^5$ HX32 tumour cells were injected i.v. into Ara-C-pretreated mice, 8/15 mice developed lung tumours.

### DISCUSSION

This preliminary report of our experience with improved methods of immune-suppression has shown that the use of 200 mg/kg of Ara-C followed 2 days later by 900 rad whole-body irradiation is a promising new technique. The cell-titration experiments (Fig. 2) indicate that when viable tumour cells were implanted in the presence of an excess of lethally irradiated cells, the mice prepared using Ara-C were at least as receptive as mice prepared using the minimum number of grafted marrow cells. We have also shown that the widely used practice of reconstituting with 5 × $10^6$ marrow cells may in part counteract the immune suppression induced by the whole-body irradiation. Reducing the marrow graft 25-fold to 2 × $10^5$ has, in our hands, given a useful improvement in transplantability, perhaps by reintroducing fewer T-cells or T-cell precursors. Both immune-suppressive techniques yielded mice that were more receptive to transplantation than athymic (nude) mice. In preliminary experiments we have found that treatment with cyclophosphamide shortly before implantation also improved transplantability but, since these experiments were performed on mice that were suboptimally suppressed, further work is necessary to confirm the value of this procedure.

The techniques of immune deprivation that we have used clearly do not confer long-lasting immune suppression. Our data (Fig. 3) indicate that there was a period of 5 weeks after immune suppression when the receptivity of the mice to grafting was good. The subsequent loss of receptivity was more marked in female than in male mice. The length of the period of high receptivity might be shorter if a more sensitive test had been used (*i.e.* a smaller inoculum of tumour cells). With a less sensitive test the period might be longer. This loss of immune suppression may be a serious problem in some types of work with xenografted tumours. It may, for instance, help to explain the low take-rate of many primary human tumour xenografts in immune-suppressed mice, if their growth rate was insufficient to beat the returning host immunity. In a series of tumour-control experiments with the HX32 tumour we have found no recurrences beyond the 6th week, as if tumours that suffered a large reduction in cell numbers were more likely to be overwhelmed by the host response. We are at present exploring methods of re-inducing the immune suppression.

The effect of lethally irradiated cells in improving the take-rate of viable tumour cells is a well-described phenomenon (Révész, 1958). It has been claimed by Peters and Hewitt (1974) to be due to the induction of a local clotting mechanism that prevents the escape of viable cells from the implantation site and their subsequent exposure to systemic host defence mechanisms. Their action in also swamping local defence mechanisms cannot be ruled out. In the present experiments the addition of lethally irradiated cells significantly improved the take-rate of the HX32 xenograft line. We are inclined to attribute our earlier failure to observe this effect with the HX18 line (Kopper and Steel, 1975) to the fact that the mice used at that time were suboptimally immune-suppressed.

The incidence of local and distant metastases in mice prepared by Ara-C protection is encouraging, and this phenomenon is now being studied in more detail. In conjunction with the higher take-rates, it leads us to believe that the Ara-C technique is a useful new method of immune suppression. By eliminating the need for a marrow graft it has the advantage of technical simplicity, and not unimportant is the fact that, in this Institute, the cost of breeding and preparing immune-suppressed mice is now about one-quarter of the cost of nude mice. Such considerations are, of course, secondary to the question of whether the mice are satisfactory hosts for the study of the growth and treatment response of human tumours, and in our judgement the choice between immune-suppressed and nude mice is from this standpoint still an open one. The present work suggests that for relatively short-term experiments, for instance those involving cell-survival measurements (Courtenay *et al.*, 1976), the immune-suppressed mice may be superior, while for long-term experiments the nude mice have the advantage.

We are grateful for the support and encouragement of Professors J. M. Peckham and L. F. Lamerton. The technical work was largely performed by Mr. J. E. Gibbs, Miss J. Mills, and Mr T. Merryweather who prepared and maintained the immune-deficient mice. The project has been greatly influenced by the work of Dr J. L. Millar and Dr N. M. Blackett, to whom we are grateful for advice on the Ara-C-protection technique.

### REFERENCES

Courtenay, V. D., Smith, I. E., Peckham, M. J. & Steel, G. G. (1976) *In vitro* and *In vivo* Radiosensitivity of Human Tumour Cells Obtained from a Pancreatic Carcinoma Xenograft. *Nature, Lond.*, **263**, 771.

HERSCH, E. M. (1973) Modification of Host Defence Mechanisms. In *Cancer Medicine* Eds. J. F. Holland and E. Frei III. Philadelphia: Lea & Febiger. p. 681.

KOPPER, L. & STEEL, G. G. (1975) The Therapeutic Response of Three Human Tumor Lines Maintained in Immune-suppressed Mice. *Cancer Res.*, **35**, 2704.

MILLAR, J. L. (1976) Protective Effect of Cyclophosphamide or Cytosine Arabinoside Pretreatment on Animals Given a Lethal Dose of Gamma Irradiation. *Expl Haematol.*, **4**, Suppl. 68 (Abstract).

MILLAR, J. L., HUDSPITH, B. N. & BLACKETT, N. M. (1975) Reduced Lethality in Mice Receiving a Combined Dose of Cyclophosphamide and Busulphan. *Br. J. Cancer*, **32**, 193.

MILLER, J. F. A. P., DOAK, S. M. A. & CROSS, A. M. (1963) Role of the Thymus in Recovery of the Immune Mechanism in the Irradiated Adult Mouse. *Proc. Soc. exp. Biol. Med.*, **112**, 785.

PETERS, L. J. & HEWITT, H. B. (1974) The Influence of Fibrin Formation on the Transplantability of Murine Tumour Cells: Implications for the Mechanism of the Révész Effect. *Br. J. Cancer*, **29**, 279.

PICKARD, R. G., COBB, L. M. & STEEL, G. G. (1975) The Growth Kinetics of Xenografts of Human Colorectal Tumours in Immune-deprived Mice. *Br. J. Cancer*, **31**, 36.

RÉVÉSZ, L. (1958) Effect of Lethally Damaged Tumor Cells upon the Development of Admixed Viable Cells. *J. natn. Cancer Inst.*, **20**, 1157.